# Practical Medicine

**Published:** 1850-10

**Authors:** 


### PRACTICAL MEDICINE.

We are preparing a series of papers on some of the principal articles used in the practice of medicine, and shall commence their publication in the November number of this *Journal*. B.

